# Development and Evaluation of a Medication Adherence Measure for Inhaler Use Among Patients with Asthma

**DOI:** 10.21315/mjms-01-2025-013

**Published:** 2025-04-30

**Authors:** Woranuch Saengcharoen, Nisanat Nanual, Sanguan Lerkiatbundit

**Affiliations:** 1 Department of Clinical Pharmacy, Faculty of Pharmaceutical Sciences, Prince of Songkla University, Songkhla, Thailand; 2 Department of Pharmacy and Consumer Protection, Somdejpraboromrachineenart Natawee Hospital, Songkhla, Thailand; 3 Department of Social and Administrative Pharmacy, Faculty of Pharmaceutical Sciences, Prince of Songkla University, Songkhla, Thailand

**Keywords:** medication adherence, measure, inhaler, asthma, development

## Abstract

**Background:**

Limited scales exist for assessing adherence among inhaler users, and information on validity and diagnostic characteristics is lacking. This study aimed to develop a Medication Adherence Measure for Inhaler Users (MAM-I) and assess its reliability, validity, and cutoff score for determining nonadherence in patients with asthma.

**Methods:**

A cross-sectional study, which included 145 patients with asthma, was conducted. The participants completed the Mini Asthma Quality of Life Questionnaire (MiniAQLQ), the Marlowe–Crowne Social Desirability Scale (MCSDS), and the 7-item MAM-I. The reliability of the scale was determined using Cronbach’s alpha, and its validity was examined in terms of criterion, concurrent, predictive, and construct validity. The diagnostic characteristics and cutoff point for nonadherence to inhaler use were also evaluated using the receiver operating characteristic (ROC) curve, with asthma control at month 6 as the gold standard.

**Results:**

The MAM-I showed a Cronbach’s alpha value of 0.784 at baseline and 0.749 at month 6. Significant correlations were found between the MAM-I scores and adherence scores (*P* < 0.001), asthma control levels (*P* < 0.001), and quality of life scores (*P* < 0.001). The cutoff point for nonadherence was < 15, with a sensitivity of 78.95%, specificity of 98.13%, positive predictive value (PPV) of 93.75%, negative predictive value (NPV) of 92.92%, and accuracy of 93.10%.

**Conclusion:**

The MAM-I shows satisfactory reliability and validity, with good diagnostic properties and a cutoff score of less than 15. This scale can be a helpful tool for identifying inhaler nonadherence in asthma management.

## Introduction

Treatment adherence improves disease control and lowers morbidity and mortality (1). Adherence to long-term therapy is defined by the World Health Organization as “the extent to which a person’s behaviour—taking medication, following a diet and/or executing lifestyle changes—corresponds with agreed recommendations from a health care provider” (2). By definition, healthcare professionals need to be aware of the extent to which patients take their prescribed medications because it impacts therapeutic outcomes.

Asthma is a common chronic disease with an increasing global burden. Inhaled corticosteroids (ICSs) are the mainstay pharmacological treatment for airway inflammation in asthma. The advantages of using ICSs include improved lung function and asthma symptoms and fewer exacerbations (3, 4). The regular use of ICSs (over 75% adherence) can prevent asthma exacerbations by 24%, resulting in a reduction in disability, hospitalisations, and fatalities, as well as an increase in quality of life (5). Despite this, patients with asthma have suboptimal adherence to ICS medications globally, ranging from 48% to 86% (5, 6). A previous study on adults with asthma reported that inadequate adherence to ICS therapy was significantly associated with lower levels of asthma control (adjusted odds ratio [aOR] = 0.18; 95% confidence interval [CI]: 0.09, 0.35; *P* < 0.001) (6). Poor asthma control results in hospital admission and often leads to rehospitalisation due to exacerbation. Asthma-related re-hospitalised patients have a substantially increased risk of eventual mortality (adjusted hazard ratio [aHR] 2.80; 95% CI: 1.95, 4.01) (7). Adherence to ICSs is a major concern in asthma treatment. To measure patients’ inhaler adherence, reliable and valid instruments are needed to better assess maintenance therapy use and promote treatment adherence.

Numerous self-report scales have been developed and are commonly used to evaluate treatment adherence in clinical settings owing to their low cost, ease of administration, and simplicity of evaluation (8). Many scales for assessing medication use in asthma capture adherence barriers or reasons for medication nonadherence accompanied by patient adherence behaviours (9–11). Moreover, several adherence measurement tools for inhaler or ICS use have been designed to examine patient beliefs about inhaler treatment (12, 13). Accordingly, the scales available to assess inhaler adherence behaviour are limited. This study aimed to develop a Medication Adherence Measure for Inhaler Users (MAM-I) and determine its reliability, validity, and cutoff score for asthma nonadherence. The resulting tool will assist healthcare providers in identifying inhaler nonadherence in patients with asthma. Healthcare providers can then thoroughly assess any drug-related problems and provide appropriate interventions.

## Methods

### Study Setting and Participants

The MAM-I was validated between August 2020 and May 2021. This cross-sectional study was conducted at a community hospital in southern Thailand.

Patients were recruited at an asthma clinic at the study site. Eligible patients were aged between 20 and 60 years, diagnosed with asthma, treated with ICS medications, and understood Thai. Those unable to complete the questionnaires because of illness or cognitive problems were excluded. The sample size for validation of the MAM-I was based on the recommendation that 2 to 20 participants per item are required (14). This scale has seven items; therefore, the required sample size ranged from 14 to 140. Convenience sampling was used for recruitment, and 145 patients with asthma were included.

### Development of the MAM-I

The MAM-I was modified from the Medication Adherence Scale for Thais (MAST) (15), an 8-item scale for assessing adherence to oral medication therapy.

Seven items of the MAST were revised to be specific to inhaler devices, and one item that did not apply to inhalers was omitted. The 7-item MAM-I is presented in the Appendix. Questions about medication adherence behaviour appear in items 1 to 5, and questions about follow-up visit attendance appear in items 6 and 7. Rating scales with fewer response options minimise confusion and reduce the burden of completing questionnaires (16). Scales with four or five options are particularly effective as they exhibit key characteristics such as hierarchical ordering (distinct and logically sequenced categories), balanced utilisation of all categories, and comprehensive representation of the underlying construct (17). Accordingly, the Likert scale was reduced from 6 to 4 points, with responses ranging from never, 1 to 5 times/month, 6 to 10 times/month, and more than 10 times/month for items 1 to 5 and never, rarely, sometimes, and often for items 6 and 7. Responses to each item are scored from 0 (never) to 3 (more than 10 times/month or often). Higher scores indicated greater adherence to inhaler use.

The items were re-examined for content validity by a panel of seven experts comprising one academic pharmacist with expertise in scale development, two physicians who were specialists in asthma, two hospital pharmacists, and two nurses with experience in asthma care for at least 5 years. The resulting scale was pretested with four patients with asthma to obtain feedback on question clarifications. The scale was then pilot tested on 30 patients.

### Data Collection Procedures

Data were collected over three consecutive medical appointments at 3-month intervals. One research pharmacist recorded the patients’ demographic and clinical information by reviewing their medical records and interviewing them. Information about asthma status was gathered, including previous history of emergency room (ER) visits and hospitalisation due to exacerbations within 3 months before visits, as well as levels of asthma control (well-controlled/not well-controlled) according to the Global Initiative for Asthma guidelines (daytime symptoms, asthmatic night waking, management with reliever, and limited activity) within 4 weeks before visits (3). Patients who did not meet all these criteria were classified as having “well-controlled asthma”. If one or more criteria were met, the condition was classified as “not well-controlled asthma”.

At each visit, the inhaler techniques, peak expiratory flow rate (PEFR) percentage, amount of medication remaining in the inhalers, and frequency of ER utilisation and hospitalisation were determined. Adherence to metered-dose inhaler use was calculated as follows: [(weight of medication received – weight of medication remaining)/weight of medication received] × 100. Adherence to Turbuhaler and Accuhaler was determined using a dose counter.

The Mini Asthma Quality of Life Questionnaire (MiniAQLQ) (18) and the Marlowe–Crowne Social Desirability Scale (MCSDS) (19) were translated from English into Thai using the translation and back-translation methods described in previous studies (20, 21). The participants completed the MiniAQLQ and the MAM-I at their first and last appointments. The MiniAQLQ contains 15 questions with four domains: symptoms, activities, emotions, and environment. The scoring range is 1 to 7, with higher scores indicating a better quality of life. The MCSDS was administered during the second appointment. The MCSDS assesses whether an individual responds to social approval or social desirability bias with positive or negative response tendencies. The scale comprises 13 questions with “yes or no” responses. A high score indicates a tendency to respond favourably to social desirability.

### Analyses

The reliability of the MAM-I was assessed by examining the item-total correlation coefficients and Cronbach’s alpha. Cronbach’s alpha was also used to evaluate the reliability of the MiniAQLQ and the MCSDS (18, 19). An item-total correlation coefficient of at least 0.3 is considered acceptable item reliability (22). A Cronbach’s alpha value of 0.7 or above is recommended for adequate internal consistency (23). The test-retest reliability of the MAM-I was assessed using the Pearson correlation coefficient of the scores measured at baseline and month 6. A correlation coefficient of 0.7 or more is interpreted as having good reliability (22).

Criterion validity was examined based on the associations between medication adherence, calculated from the medicine remaining in the inhalers, and the MAM-I scores. Moreover, multivariate logistic regression was performed to assess the criterion validity of the MAM-I or its predictability of future ER visits while controlling for patients’ sex, smoking status, period of asthma diagnosis, and number of correct inhaler usage steps. Concurrent and predictive validity analyses were performed based on the associations of PEFR, asthma control, and ER visits with the MAM-I scores. Construct validity was assessed by analysing the associations of the MiniAQLQ and MCSDS scores with the MAM-I scores. This approach helped determine whether the MAM-I accurately measures medical adherence in accordance with theoretical expectations. Known-group validity was assessed through the ability of the MAM-I to distinguish between those with well-controlled asthma vs those with poor asthma control, and those with no ER visits vs those with ER visits. The Pearson correlation coefficients were used in all validity tests. Independent sample t-tests were performed to evaluate known-group validity and assess concurrent and predictive validity related to asthma control and ER visits.

Diagnostic properties were estimated by receiver operating characteristic (ROC) curve analysis, using asthma control as the gold standard. The cutoff point for judging nonadherence was defined as the MAM-I scores where the sum of sensitivity and specificity showed the highest values. Sensitivity, specificity, positive and negative predictive values (PPV and NPV, respectively), positive and negative likelihood ratios (LR+ and LR−, respectively), and areas under the curve (AUCs) were calculated to show diagnostic characteristics of the MAM-I. A *P*-value < 0.05 was considered statistically significant. Statistical analyses were conducted using Statistical Package for the Social Sciences (SPSS) version 22 (IBM Corp., Armonk, NY, US).

## Results

### Patient Characteristics

Of the 145 patients who completed the follow-up, 77.9% were female (Table 1). The patients had a mean age of 48.63 (standard deviation = 9.89) years, and 59.3% completed primary school. In 46.9% of the participants, it had been less than 10 years since their asthma diagnosis, and 88.3% had no ER visits due to disease exacerbation in the previous 6 months.

### Reliability

The item-total correlation coefficients of the MAM-I measured at baseline and month 6 were at least 0.3 (ranging from 0.300 to 0.680).

The MAM-I had Cronbach’s alpha values of 0.784 at baseline and 0.749 at month 6, whereas the MiniAQLQ had Cronbach’s alpha values of 0.929 and 0.936 at the same time points, respectively. The MCSDS had a Cronbach’s alpha of 0.840 at month 3. The Cronbach’s alpha for the MAM-I did not increase substantially with the exclusion of any items; hence, all seven items were retained. Furthermore, the test-retest reliability of the scale was 0.817 (*P* < 0.001).

### Validity

The correlations between the MAM-I scores and several variables are presented in Table 2. Regarding criterion validity, the MAM-I scores were significantly and positively correlated with the inhaler adherence scores at baseline, month 3, and month 6 (*r* = 0.494 to 0.713; *P* < 0.001). Higher MAM-I scores were associated with greater inhaler adherence. Multivariate logistic regression was employed to assess the validity of the MAM-I and its predictability for future ER visits (Table 3). Patient sex, smoking status, period of asthma diagnosis, and number of correct steps in inhaler use demonstrated by patients were not significantly associated with ER visits. However, increased MAM-I scores were significantly associated with a reduced risk of visiting the ER in the following 6 months (aOR = 0.29; 95% CI: 0.16, 0.53; *P* < 0.001), confirming the criterion validity of the scale.

Non-significant correlations between the MAM-I and PEFR values were identified at baseline, month 3, and month 6 (*P* > 0.05) (Table 2). However, analyses of concurrent and predictive validity revealed that patients with well-controlled asthma or no ER visits had significantly higher MAM-I scores than those of patients with inadequately controlled asthma or ER visits at all three study points (*P* < 0.001) (Table 4). These findings demonstrate the concurrent and predictive validity of the MAM-I.

The MAM-I demonstrated known-group validity by significantly distinguishing between groups based on asthma control status and ER utilisation patterns (*P* < 0.001) as shown in Table 4. The findings of the construct validity test are presented in Table 2, indicating significant positive relationships between the MAM-I and MiniAQLQ scores (*P* < 0.001) at baseline and month 6. No significant relationship was observed between the MAM-I and MCSDS scores (*P* = 0.856). The results indicated that greater MAM-I scores were associated with a higher quality of life but not a tendency to respond based on social desirability. These results provide evidence supporting the construct validity of the MAM-I.

### ROC Curve Analysis

The ROC curves were constructed for the MAM-I scores using asthma control at baseline, month 3, and month 6 (well-controlled or not well-controlled) as the gold standards. The scale had AUCs of 0.921, 0.917, and 0.919 at the three time points, respectively. A cutoff of 15 when using asthma control at baseline and month 6 as the gold standard resulted in high sensitivity and specificity as well as the highest sum of both values. When asthma control at month 3 was used as the gold standard, the cutoff point was close to 15. Therefore, 15 was decided as the MAM-I cutoff point. MAM-I scores of < 15 represented nonadherence to inhaler use.

At the optimal cutoff point, the MAM-I showed sensitivity ranging from 70.73% to 87.87% and specificity ranging from 97.11% to 98.13% (Table 5). Using asthma control at month 6 as the gold standard, the probability of patients with inadequately controlled asthma being nonadherent to medication was 78.95%. In contrast, the probability of patients with well-controlled asthma adhering to medication was 98.13%. The PPV and NPV of the scale were 90.63% to 93.75% and 89.38% to 96.46%, respectively. Concerning the PPV, 93.75% of patients who were nonadherent were likely to have inadequately controlled asthma in the next 6 months. The NPV showed that 92.92% of patients who were adherent had a chance of having well-controlled asthma in the following 6 months. The LR+ and LR− of the MAM-I were 24.52 to 42.24 and 0.12 to 0.30, respectively in the 6-month interval. Patients with inadequately controlled asthma were 42.24 times more likely to be nonadherent than those with well-controlled asthma. In contrast, patients with inadequately controlled asthma were 0.21 times as likely as those with well-controlled asthma to be adherent. The accuracy of the scale ranged from 89.66% to 95.17%. Accordingly, the MAM-I scale was a satisfactory predictor of inhaler adherence with high PPV, NPV, LR+, and low LR− values. In addition, the evaluation of asthma control at baseline, month 3, and month 6 demonstrated that patients with insufficient adherence to inhaler medication (MAM-I scores < 15) had a significantly lower proportion of well-controlled asthma than the proportion in those with sufficient adherence (MAM-I scores ≥ 15), with *P* < 0.001 at all time points (Table 5).

## Discussion

The current study presents the development of the MAM-I, a new self-administered questionnaire on inhaler use adherence. The MAM-I demonstrated adequate reliability, validity, and good diagnostic characteristics, including sensitivity and specificity, for assessing inhaler adherence in patients with asthma.

The MAM-I had an acceptable reliability, with item-total correlation coefficients above 0.3 (0.300 to 0.680) and Cronbach’s alpha values above 0.7 (0.749 to 0.784) (22, 23). Its internal consistency is comparable to those of other inhaler adherence scales measuring beliefs or behaviours in patients with asthma, with Cronbach’s alpha values ranging from 0.70 to 0.80 (13, 24, 25). Good test-retest reliability was achieved with the MAM-I which is comparable to the Test of the Adherence to Inhalers (TAI) in asthma with a value of 0.883 (26). The reliability of a scale is affected by various factors, including participant characteristics, testing procedures, and the length and difficulty of testing content (27). Furthermore, the number of response levels for each item influences scale reliability (28). Although the MAM-I scale has fewer response options per item (four instead of six) than the original MAST scale, its reliability was acceptable. This simplifies the answering process while potentially increasing the accuracy of the results.

Electronic monitoring devices as an objective method are recommended as the gold standard for investigating adherence to inhalation therapy. The adoption of electronic monitors was limited, most likely because of their high cost and inconvenience (29). In this study, adherence was measured objectively by weighing inhaler canisters and comparing the results with self-reported MAM-I scores. A strong correlation was found between the changes in canister weight and the MAM-I scores. This correlation implies the criterion validity of the MAM-I. Similarly, studies of adherence to inhaled medications using the TAI and the Medication Adherence Report Scale (MARS) in asthma or chronic obstructive pulmonary disease (COPD) reported that scores from the test had a significant correlation with electronic monitors (*P* ≤ 0.01) (25, 26). Participants with higher MAM-I scores had significantly better asthma control or no ER utilisation than those with lower MAM-I scores. This finding supports the concurrent and predictive validity of the MAM-I. A systematic review by Chongmelaxme et al. (30) confirmed that higher adherence to asthma medications leads to better disease control. This systematic review indicated that patients with 80% or higher adherence lowered the odds of asthma exacerbations by 47% compared with those with less than 80% adherence. However, adherence of 20% to 49% was not associated with a reduction in exacerbations (30). Increased adherence to asthma medications results in a substantial decline in exacerbations. Positive relationships between the MAM-I and quality of life scores, as measured by the MiniAQLQ (16), were observed in this study. According to previous research, adherence is related to quality of life (*r* = 0.14; *P* = 0.035) and symptom control (*r* = 0.23; *P* < 0.001) (31). Additionally, a systematic review indicated that the cost-effectiveness of asthma treatment is influenced by the level of adherence. This suggests that full adherence is cost-effective (32). For nonadherent patients, healthcare professionals may consider providing educational interventions to promote continuous medication use, which may improve clinical outcomes and lower healthcare costs (5).

The optimal MAM-I score for determining inhaler nonadherence was less than 15, with an AUC of 0.919. An AUC greater than 0.8 is classified as high accuracy. In accordance with the high AUC, the MAM-I showed a good ability to distinguish between those with and without adherence to inhaler use (33). In the current study, the sensitivity and specificity of the MAM-I were 78.95% and 98.13%, respectively, which were superior to those of the MARS (sensitivity 60% and specificity 71%) and the Inhaler Adherence Questionnaire (IAQ) (sensitivity 73% and specificity 80%) in patients with asthma treated with inhalation (24, 25). According to these findings, the MAM-I may be a better tool for identifying patients who do not adhere to their inhaler medication regimen than the MARS and IAQ, which are commonly used tools for measuring inhaler adherence. In addition, the PPV and NPV of the MAM-I were 93.75% and 92.92%, respectively, which were higher than those of the TAI (48.8% and 63.2%, respectively) (26). The MAM-I predicts true positive and true negative cases of nonadherence better than the TAI. This has significant potential implications for clinical decision-making, as healthcare providers can use the MAM-I to identify patients who may benefit from additional support or interventions to improve their medication adherence.

The strengths of this study were the comprehensive assessment of the MAM-I in terms of its reliability, validity, and diagnostic characteristics for determining medication adherence. The validity of the scale was assessed using several approaches, including criterion, concurrent, predictive, and construct validity. Furthermore, the ROC curve was used to determine the cutoff point for nonadherence with high sensitivity and specificity, indicating good diagnostic properties of the MAM-I.

The current study has some limitations. First, all the participants were patients attending an asthma clinic in a single study setting in a developing country. This may limit the generalisability of the findings. Second, this study used the MAM-I to assess adherence to inhaled medications only in patients with asthma and not in those with COPD or other diseases treated with inhalers. Further studies are warranted to evaluate this scale in patients with COPD and other diseases. Third, no electronic monitors were used as reference standards for the MAM-I scores. However, the inhaler canister weights were used instead. Lastly, the MAM-I was only tested in Thai, and it is necessary to translate and test the scale in multiple languages.

## Conclusion

The results of this study show that the MAM-I is a reliable and valid scale for assessing inhaler adherence in patients with asthma. The cutoff point for nonadherence (< 15) had high sensitivity, specificity, PPV, NPV, and accuracy. This indicates that the MAM-I can effectively identify patients who do not adhere to their inhaler medication regimens. The MAM-I can be used in clinical settings and has the potential to improve clinical care in patients with asthma by facilitating accurate assessment and monitoring of inhaler adherence.

## Figures and Tables

**Table 1:** Demographic and clinical characteristics of participants (*n* = 145)

Variable	*n* (%)	Mean (SD)
Sex		
Male	32 (22.1)	
Female	113 (77.9)	

Age (years)		48.63 (9.89)

Education level		
Primary school	86 (59.3)	
Secondary school	36 (24.8)	
College or higher	23 (15.9)	

Duration of asthma (years)		
< 10	68 (46.9)	
10–20	31 (21.4)	
> 20	46 (31.7)	

History of ER visits with exacerbated asthma in the previous 6 months		
None	128 (88.3)	
1 or 2 visits	17 (11.7)	

Current ICS use		
Budesonide (MDI)	38 (26.2)	
Salmeterol + fluticasone (MDI)	37 (25.5)	
Budesonide + formoterol (Turbuhaler)	49 (33.8)	
Salmeterol + fluticasone (Accuhaler)	21 (14.5)	

Notes: SD = standard deviation; MDI = metered-dose inhaler

**Table 2:** Correlations between the MAM-I scores (at baseline) and validated indicators (Pearson correlation coefficient) (*n* = 145)

Indicator	Correlation coefficient with the MAM-I scores	*P*-value
Criterion validity		
Inhaler adherence (%)		
Baseline	0.713	< 0.001
Month 3	0.510	< 0.001
Month 6	0.494	< 0.001
PEFR (%)		
Baseline	0.020	0.815
Month 3	0.009	0.913
Month 6	0.037	0.657
Construct validity		
MiniAQLQ		
Baseline	0.412	< 0.001
Month 6	0.403	< 0.001
MCSDS		
Month 3	−0.015	0.856

**Table 3:** Multivariate logistic regression analysis of ER utilisation (in the next 6 months) (*n* = 145)

Variable	Adjusted OR	95% CI	*P*-value
Sex			
Male	Reference		
Female	2.20	0.12, 41.37	0.597
Smoking status			
No smoking	Reference		
Smoking	6.24	0.11, 360.92	0.377
Period of asthma diagnosis (years)			
< 10	Reference		
10–20	7.27	0.82, 64.36	0.075
21–30	0.10	0.01, 4.86	0.248
> 30	0.31	0.02, 4.86	0.405
The number of correct steps in inhaler use	0.40	0.11, 1.44	0.161
MAM-I scores measured at baseline	0.29	0.16, 0.53	< 0.001

Note: OR = odds ratio

**Table 4:** Association of the MAM-I scores (measured at baseline) with asthma control and ER utilisation (measured at baseline, month 3, and month 6) (*n* = 145)

Measuring times for outcomes	Outcome	MAM-I scores at baseline^a^		Mean difference (95% CI)	*P*-value

		*n*	Mean (SD)		
	Asthma control^a^ within 4 weeks before visits				

Baseline	Well-controlled asthma	112	17.96 (1.97)	7.86 (6.04, 9.69)	< 0.001
	Not well-controlled asthma	33	10.09 (5.06)		

Visit at month 3	Well-controlled asthma	104	18.14 (1.91)	7.00 (5.37, 8.62)	< 0.001
	Not well-controlled asthma	41	11.15 (5.03)		

Visit at month 6	Well-controlled asthma	107	18.09 (1.86)	7.36 (5.66, 9.05)	< 0.001
	Not well-controlled asthma	38	10.74 (5.05)		

	ER utilisation within 3 months before visits				

Baseline	No ER visits	40	19.65 (1.64)	4.81 (3.81, 5.81)	< 0.001
	ER visits	105	14.84 (4.44)		

Visit at month 3	No ER visits	67	19.13 (1.43)	5.52 (4.44, 6.60)	< 0.001
	ER visits	78	13.62 (4.55)		

Visit at month 6	No ER visits	59	19.24 (1.34)	5.18 (4.14, 6.22)	< 0.001
	ER visits	86	14.06 (4.58)		

Notes: SD = standard deviation;

a Range: 0–21

**Table 5:** Characteristics of the MAM-I (at baseline) to predict asthma control at a cutoff point of 15 (*n* = 145)

The MAM-I (at baseline)	Asthma control		

	Baseline	Month 3	Month 6
Sensitivity	87.87	70.73	78.95
Specificity	97.32	97.11	98.13
PPV	90.63	90.63	93.75
NPV	96.46	89.38	92.92
LR+	32.81	24.52	42.24
LR−	0.12	0.30	0.21
Accuracy	95.17	89.66	93.10

The MAM-I scores	Well-controlled asthma [*n* (%)]		

	Baseline	Month 3	Month 6

Cutoff point of 15			
< 15	3 (2.7)	3 (2.9)	2 (1.9)
≥ 15	109 (97.3)	101 (97.1)	105 (98.1)

*P*-value < 0.001 < 0.001 < 0.001

## Appendix

**Table:** Items of the MAM-I

Item		Frequency			
1.	In the last month, how frequently did you forget to use the inhaler (missed some doses)?	> 10 times/month	6–10 times/month	1–5 times/month	Never
2.	In the last month, how frequently did you change the dose of the inhaler to meet your needs (i.e., used more or less than you should)?	> 10 times/month	6–10 times/month	1–5 times/month	Never
3.	In the last month, how frequently did you stop using the inhaler by yourself?	> 10 times/month	6–10 times/month	1–5 times/month	Never
4.	In the last month, how frequently did you use the inhaler at the wrong time (more than 1 hour before or after the usual time)?	> 10 times/month	6–10 times/month	1–5 times/month	Never
5.	In the last month, how frequently did you not complete all doses of your inhaler, for example, forgetting to use the inhaler or forgetting to bring it to work during the day, or forgetting to bring it on a long trip?	> 10 times/month	6–10 times/month	1–5 times/month	Never
6.	How frequently did you fail to attend a doctor’s visit (missed or rescheduled a doctor’s appointment)?	Often	Sometimes	Rarely	Never
7.	How frequently did you skip using the inhaler because you did not attend your doctor’s appointment on time?	Often	Sometimes	Rarely	Never
